# A Calixarene Assembly Strategy of Combined Anti-Neuroinflammation and Drug Delivery Functions for Traumatic Brain Injury Therapy

**DOI:** 10.3390/molecules27092967

**Published:** 2022-05-06

**Authors:** Chunxiao Wang, Yu-Xuan Chang, Xi Chen, Lihuan Bai, Heping Wang, Yu-Chen Pan, Chunqiu Zhang, Dong-Sheng Guo, Xue Xue

**Affiliations:** 1 Laboratory of Theranostical Nanomedicine, State Key Laboratory of Medicinal Chemical Biology, College of Pharmacy, Nankai University, Haihe Education Park, 38 Tongyan Road, Tianjin 300353, China; 1120170448@mail.nankai.edu.cn (C.W.); 2120201154@mail.nankai.edu.cn (X.C.); 18737620279@163.com (L.B.); hepingwang@mail.nankai.edu.cn (H.W.); zhangcq@nankai.edu.cn (C.Z.); 2 Key Laboratory of Functional Polymer Materials (Ministry of Education), State Key Laboratory of Elemento-Organic Chemistry, College of Chemistry, Nankai University, 94 Weijin Poad, Tianjin 300071, China; yxchang@sas.upenn.edu (Y.-X.C.); panyuchen@mail.nankai.edu.cn (Y.-C.P.)

**Keywords:** calixarenes, self-assembly, neuroinflammation, microglia, traumatic brain injury

## Abstract

Excessive inflammatory reaction aggravates brain injury and hinders the recovery of neural function in nervous system diseases. Microglia, as the major players of neuroinflammation, control the progress of the disease. There is an urgent need for effective non-invasive therapy to treat neuroinflammation mediated by microglia. However, the lack of specificity of anti-inflammatory agents and insufficient drug dose penetrating into the brain lesion area are the main problems. Here, we evaluated a series of calixarenes and found that among them the self-assembling architecture of amphiphilic sulfonatocalix[8]arene (SC8A12C) had the most potent ability to suppress neuroinflammation in vitro and in vivo. Moreover, SC8A12C assemblies were internalized into microglia through macropinocytosis. In addition, after applying the SC8A12C assemblies to the exposed brain tissue, we observed that SC8A12C assemblies penetrated into the brain parenchyma and eliminated the inflammatory factor storm, thereby restoring neurobiological functions in a mouse model of traumatic brain injury.

## 1. Introduction

Neuroinflammation, which leads to neurological dysfunction [1], glial scar [2] formation, and demyelination [3], universally occurs in various neurological disorders, including Parkinson’s syndrome [4], stroke [5], and traumatic brain injury (TBI) [6]. As the resident immune cells in the brain, microglia are the first to sense lesions but are easily over-activated into a destructive state to cause aggravated development of an inflammatory environment [7,8,9,10,11]. At present, there is a dearth of drugs targeting neuroinflammation that are usable in clinical settings. This is because of the complex pathological mechanisms involved. Nonsteroidal anti-inflammatory drugs (NSAIDs) reduce the production of pro-inflammatory prostaglandin to relieve pain, but as a kind of broad-spectrum anti-inflammatory drug, they cannot provide targeted treatment for neuroinflammation [12]. In addition, due to the existence of the blood-brain barrier (BBB), the dose of drugs targeting the damaged brain is reduced by non-invasive administration, which limits the development of drugs.

TBI is a mechanical brain injury that exposes brain tissue, often caused by combat, falls, traffic accidents, and sports activities, which have fairly high morbidity and mortality worldwide [13]. The profound neuroinflammatory response triggered by TBI leads to the rupture of the BBB. Only local administration can be used for first aid, because this way of administration avoids the difficulty of drugs entering brain tissue through blood flow. Therefore, it is necessary to explore novel active ingredients to suppress microglia over-activation and local application.

Over the past decades, increasing studies have shown that supramolecular chemistry is widely used in the biomedical field due to its advantages, such as simplicity, low cost, molecular-level control of composition, and quantitative drug loading [14,15]. Calixarenes, a new generation of supramolecular compounds, provide unlimited possibilities for their application in the biomedical field because of their adjustable skeleton, various modifications, and self-assembly characteristics [16]. The potential for biomedical applications benefits from the rich chemical structure of calixarene. Yitzchaik et al. found that p-sulfonatocalix[6]arene (SC6A) was the most sensitive sensing system for acetylcholine in their experiment with SC(4,6,8)A as hosts and trans-4-[4-(dimethylamino)styryl]-1-methylpyridinium-p-toluenesulfonate (4ASP) as an indicator [17]. Hennig and us constructed a phosphorylation-responsive membrane transport system and found that amphiphilic calixarene had higher efficiency in transporting cell-penetrating peptides than hydrophilic calixarene [18]. Furthermore, the introduction of lower-rim alkylation on calixarene to maintain the cone conformation is important for inhibiting human immunodeficiency virus (HIV) and hepatitis C virus (HCV) infections [19]. Therefore, the structure of calixarene affects its biological activity. Thus far, calixarenes have been initially applied to nervous system disease and have shown the ability to reduce the number of plaque-associated microglia in Alzheimer’s disease [20]. However, their potential as anti-inflammatory agents has yet to be fully evaluated.

In this work, we explored the inhibitory effect of calixarenes on neuroinflammation by investigating the structure–activity relationship between calixarenes and neuroinflammation and demonstrated that the calixarene assembly amphiphilic sulfonatocalix[8]arene (SC8A12C) presents excellent anti-inflammatory effects. Then we assessed the ability of SC8A12C to alleviate lipopolysaccharide (LPS) and TBI-induced neuroinflammation in vitro and in vivo. Results from this research demonstrated that SC8A12C was internalized by microglia and produced outstanding therapeutic efficacy against neuroinflammation. Furthermore, SC8A12C was delivered to the brain parenchyma by the local administration, realizing the inhibition of neuroinflammation and neuroprotection in a mouse model of TBI. Our work provides a new perspective for the design of molecules with anti-neuroinflammatory activities.

## 2. Results

### 2.1. Study on Anti-Inflammatory Activity of Calixarene with Different Skeleton

**Figure 1:**
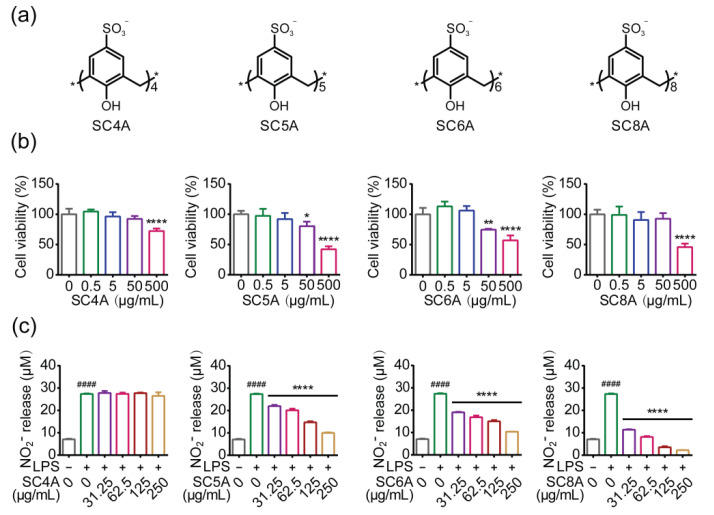
(**a**) Structure of SC4A, SC5A, SC6A, and SC8A; (**b**) viabilities of BV2 cells treated with SC4A, SC5A, SC6A, SC8A for 24 h (* *p* < 0.05, ** *p* < 0.01, **** *p* < 0.0001); (**c**) measurement of NO generation by Griess assay. BV2 cells culture medium was added with LPS (0.1 μg/mL) alone, LPS (0.1 μg/mL) with SC4A, SC5A, SC6A, SC8A for 24 h, respectively. Cells cultured without LPS (0.1 μg/mL) and calixarenes served as the control group. ^####^ *p* < 0.0001 vs. the control group; **** *p* < 0.0001 vs. the LPS group.

We employed four types of calixarenes to systematically explore the effect of calixarenes on neuroinflammatory inhibition, the structures of which are shown in Figure 1a. Prior to the structure–activity study, we tested the potential neurotoxicity of the four types of calixarenes and found that sulfonatocalix[4]arene (SC4A) and sulfonatocalix[8]arene (SC8A) exhibited relatively low toxicity with nontoxic concentration below 500 μg/mL in a BV2 microglial cell line. However, the toxic doses of sulfonatocalix[5]arene (SC5A) and sulfonatocalix[6]arene (SC6A) were below 50 μg/mL (Figure 1b). Then we used LPS-induced BV2 microglia as a typical in vitro neuroinflammatory model and determined the level of nitric oxide (NO) release as a marker to evaluate the severity of inflammation (Figure 1c). All four types of calixarenes reduced LPS-induced NO production, but SC8A markedly had the most potent effect. We chose SC8A as the preferred candidate because it exhibited both good efficacy and biocompatibility for neuroinflammatory inhibition. Calixarenes with different scaffolds show different inhibitory effects on neuroinflammatory responses. The anti-inflammatory activities were ordered: SC8A > SC6A > SC5A > SC4A, from which we conclude that the skeleton flexibility was important for anti-inflammatory.

### 2.2. Characteristics of SC8A12C Assemblies

**Figure 2:**
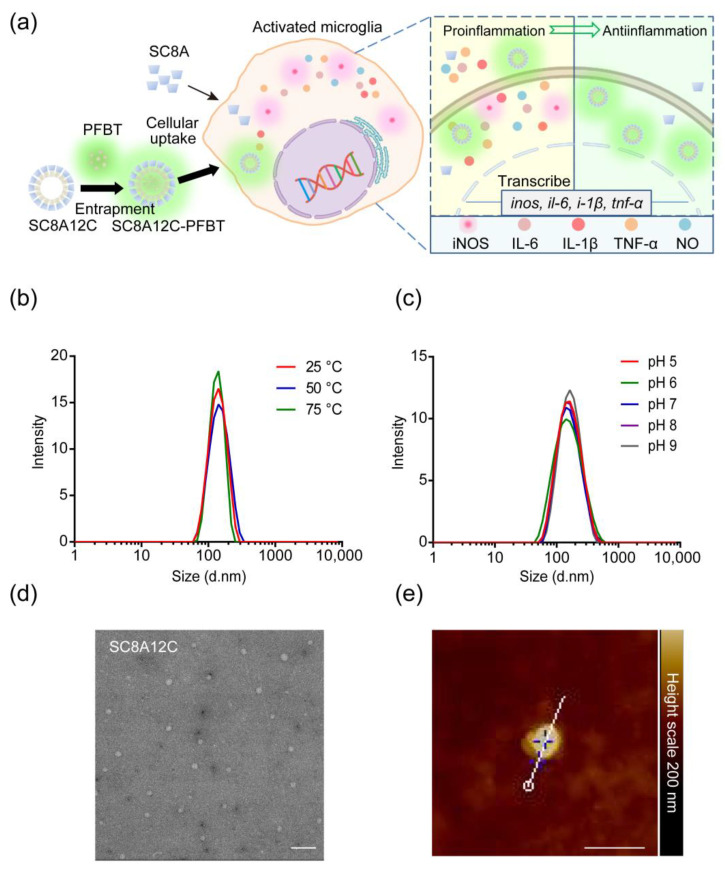
(**a**) Schematic diagram of SC8A or SC8A12C assemblies for inflammatory treatment. SC8A or SC8A12C-PFBT entered microglia to inhibit the expression of iNOS and the release of inflammatory mediators; (**b**) effect of temperature on the size of SC8A12C assemblies (25 μg/mL) in water; (**c**) effect of pH on the size of SC8A12C assemblies (25 μg/mL) in water; (**d**) TEM image of SC8A12C assemblies. Scale bar, 500 nm; (**e**) AFM image of SC8A12C assemblies. Scale bar, 500 nm.

According to our previous studies [21,22], calixarene amphiphilic molecules that form tight assemblies with alkyl chains may affect interactions between calixarenes and cell membranes, which is conducive to cell uptake. To investigate whether the assembly ability of calixarene amphiphilic molecules is important for the anti-inflammatory effect, an amphiphilic self-assembling architecture of SC8A called amphiphilic sulfonatocalix[8]arene (SC8A12C) was introduced for comparison with SC8A (Figure 2a, Figures S1 and S2). The average hydrodynamic diameter of these self-assemblies was 159.9 ± 13.7 nm (Figure 2b,c). When the ambient temperature and pH changed in a moderate range, the particle size did not change significantly, which indicated that SC8A12C assemblies had satisfactory stability. The zeta potential value of SC8A12C assemblies was −42.0 ± 2.8 mV, which demonstrated that there were sulfonic acid groups on the surface of the assemblies. It is generally believed that when the absolute value of the zeta potential is greater than 20 mV, the colloidal particles are stable, which supports that SC8A12C assemblies had excellent dispersion stability in aqueous media. Transmission electron microscopy (TEM) imaging revealed the spherical structure of the SC8A12C assemblies (Figure 2d). In aqueous media, a hydration layer was formed outside the assemblies, which caused the particle size estimated by TEM to be smaller than that measured with DLS. The microstructure information of the assemblies provided by the atomic force microscope (AFM) image proved that the micelle was the most likely structure of the assembly (Figure 2e).

### 2.3. The Anti-Inflammatory Effect of a Self-Assembling Architecture of SC8A12C

**Figure 3:**
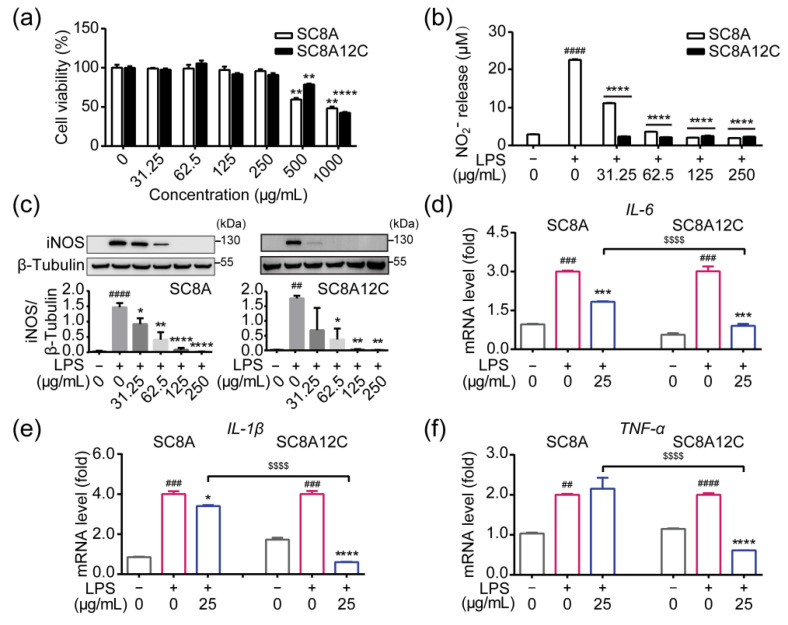
(**a**) Viabilities of BV2 cells treated with SC8A, SC8A12C for 24 h (** *p* < 0.01, **** *p* < 0.0001). (**b**) Measurement of NO generation by Griess assay. BV2 cells culture medium was added with LPS (0.1 μg/mL) alone, or LPS with SC8A, SC8A12C for 24 h, respectively. Cells cultured without LPS and calixarenes served as the control group. (**c**) Representative and quantitative Western blot results, in which the lanes represent the iNOS or β-tubulin level in the cell sample as shown above, illustrating that SC8A and SC8A12C significantly attenuated LPS-induced increases in iNOS expression. (**d**–**f**) RT-qPCR analysis proving the inhibitory effect of SC8A, SC8A12C on the transcription of LPS-induced proinflammatory genes in BV2 cells. ^##^ *p* < 0.01, ^###^ *p* < 0.001, ^####^ *p* < 0.0001 vs. the control group; * *p* < 0.05, ** *p* < 0.01, *** *p* < 0.001, **** *p* < 0.0001 vs. the LPS group; ^$$$$^ *p* < 0.0001 vs. the SC8A group.

The cytotoxicity of SC8A and SC8A12C was determined by MTT assay. There was no significant difference between SC8A and SC8A12C in the cytotoxic test (Figure 3a). However, SC8A12C displayed superior blocking of LPS-induced NO release compared with SC8A, especially with the treatment of lower dosages (Figure 3b). Nitric oxide synthase (iNOS) catalyzes L-arginine to produce NO. The inflammatory environment induces the upregulation of the iNOS level, resulting in the excessive production of NO. Western blot assessments demonstrated that iNOS expression was markedly augmented after LPS stimulation (Figure 3c), and SC8A12C treatment clearly decreased the expression level of iNOS. To further verify the anti-inflammatory effects of SC8A12C, we next examined the transcriptional level of pro-inflammatory genes including interleukin-6 (IL-6), interleukin-1β (IL-1β), and tumor necrosis factor-α (TNF-α) in a BV2 microglial cell line. Both SC8A and SC8A12C inhibited the transcription of these inflammation-related genes, but the effect of SC8A was less than that of SC8A12C, consistent with the above results (Figure 3d–f). Therefore, we speculate that the self-assembling architecture of calixarene with an alkyl chain may be the key factor contributing to the enhanced anti-inflammatory effects of SC8A12C. Therefore, we conducted an in-depth study of the SC8A12C.

### 2.4. Cellular Uptake of SC8A12C Assemblies

**Figure 4:**
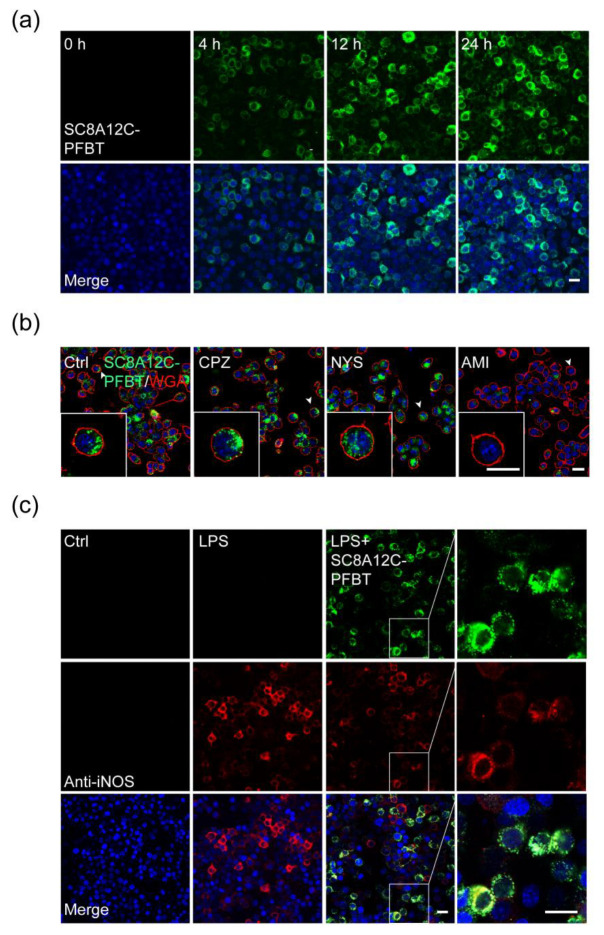
(**a**) Intracellular SC8A12C-PFBT (green) was observed in BV2 (treated with 25 μg/mL) by fluorescence imaging. Scale bar, 10 μm; (**b**) the images of SC8A12C assemblies’ internalization in BV2 cells with the treatment of CPZ (5 μM), NYS (10 μM), and AMI (0.01 μM). Scale bar, 20 μm; (**c**) colocalization detection of SC8A12C-PFBT (green) and iNOS (red). SC8A12C-PFBT located inflammatory cells and achieved an anti-inflammatory effect. Scale bar, 10 μm.

The self-assemblies formed by amphiphilic calixarenes provide a macrocyclic cavity and micelle interior, which enable drug solubilization. Drugs can either be complexed into macrocyclic cavities, be solubilized into micelles self-assemblies, or both, suggesting the potential application of amphiphilic calixarenes as drug delivery systems [23]. To investigate the drug-loading capacity of amphiphilic calixarenes, we co-assembled SC8A12C with a fluorescent probe, poly[(9,9-dioctylfluorenyl-2,7-diyl)-alt-(benzo[2,1,3]thiadiazol-4,7-diyl)] (PFBT) to form SC8A12C-PFBT and observed the cell uptake of the SC8A12C assemblies (Figure 2a). We examined the effect of SC8A12C-PFBT on NO production at the non-toxic concentration (Figure S3), and the results suggested that the loading of PFBT into the cavity of SC8A12C did not affect the inhibitory effect on LPS-induced NO production in BV2 (Figure S4). Meanwhile, the inhibitory effects of SC8A12C-PFBT on iNOS protein expression, as well as the mRNA transcription levels of inflammation-related genes, were consistent with the above findings of SC8A12C (Figures S5–S8). Notably, we found that the cellular uptake reached a peak at 12 h after SC8A12C-PFBT treatment and maintained considerably high fluorescent accumulation for at least 24 h (Figure 4a). Then, we studied the internalization mechanism of the SC8A12C assemblies (Figure 4b). Fluorescence imaging demonstrated that amiloride, a macropinocytosis inhibitor, restricted the internalization of SC8A12C. However, clathrin-mediated endocytosis inhibitors (chlorpromazine) and caveolin-mediated endocytosis inhibitors (nystatin) had no effect on cell uptake. We found that macropinocytosis played a crucial role in the uptake of SC8A12C assemblies in a microglia cell line. In addition, most iNOS-positive cells were co-localized with the SC8A12C-PFBT positive cells (Figure 4c). Accordingly, these results indicated that SC8A12C was prone to self-assembly, and these assemblies with nano-size are capable of either permeating, accumulating, or both, into the microglia to suppress neuroinflammation; the cell uptake of SC8A12C loaded with PFBT also showed that the SC8A12C assemblies are capable of drug loading and drug delivery.

### 2.5. Penetration of SC8A12C into Brain Parenchyma in a Mouse Model of TBI

**Figure 5:**
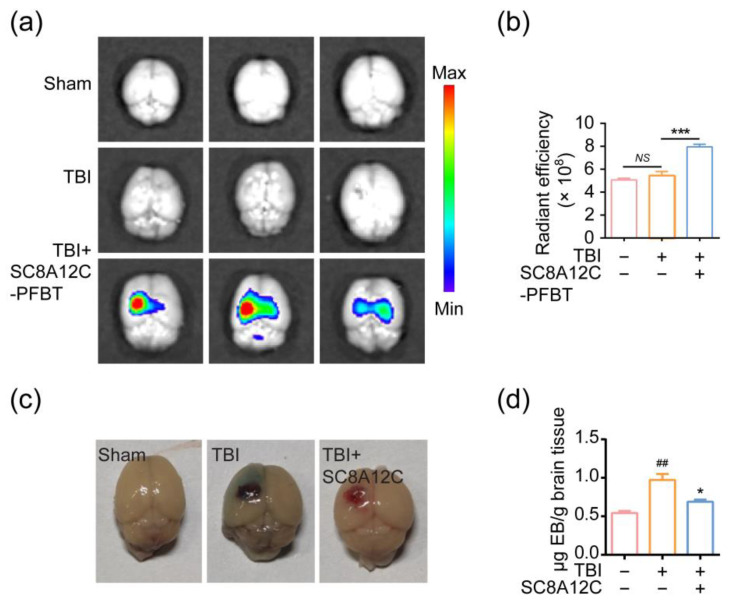
(**a**) Fluorescence imaging of the brain was performed by IVIS system; (**b**) the radiant efficiency in panel a; (**c**) EB staining imaging; and (**d**) quantitative analysis of the content of EB dye by UV spectrophotometry, respectively (*n* = 3). ^##^ *p* < 0.01 vs. sham group; * *p* < 0.05, *** *p* < 0.001 vs. TBI group, NS: No significance.

Inspired by the in vitro effects of SC8A12C against LPS-induced neuroinflammation, we next performed an in vivo study on SC8A12C. TBI refers to soft tissue, skull, and brain tissue injuries, often caused by combat, falls, traffic accidents, and sports activities, which have fairly high morbidity and mortality worldwide [13]. The primary injury rapidly stimulates microglial cells to a hyperinflammatory state, which further produces a fulminant cytokine storm that eventually causes irreversible brain damage. Therefore, targeting neuroinflammation is an excellent strategy for treating TBI [24,25,26]. However, a rare approach is currently managed in the immediate intervention after primary insults. Locally delivered agents must be developed as a practical strategy for emergency rescue to avoid the difficulty of crossing the blood-brain barrier (BBB). We administered SC8A12C to the exposed brain tissue with an eyedropper. To estimate the brain level of SC8A12C, we performed a fluorescence analysis to observe the SC8A12C-PFBT fluorescence in the mouse brain at 24 h of administration (Figure 5a,b). We found that the assemblies penetrated into the brain parenchyma. We next explored the potential therapeutic effects of SC8A12C on BBB recovery, inflammatory inhibition, and functional recovery. The inflammatory response associated with TBI results in the damage of the BBB [27]; hence, the repair of the BBB is likely to improve neurological function and brain recovery [28]. Evans Blue (EB) staining was used to detect the damage degree of the BBB [29]. The BBB permeability of mice in a TBI group was significantly increased, which indicated cerebrovascular injury had occurred (Figure 5c). Surprisingly, a single administration of SC8A12C remarkably decreased EB extravasation, which was in accordance with the quantification analysis in different groups (Figure 5d). The above results demonstrate that calixarene assemblies have the ability to penetrate deeply into the brain parenchyma, which proves that they have the potential to exert biological function in the brain.

### 2.6. In Vivo Therapeutic Effect of SC8A12C Assemblies on Traumatic Brain Injury

**Figure 6:**
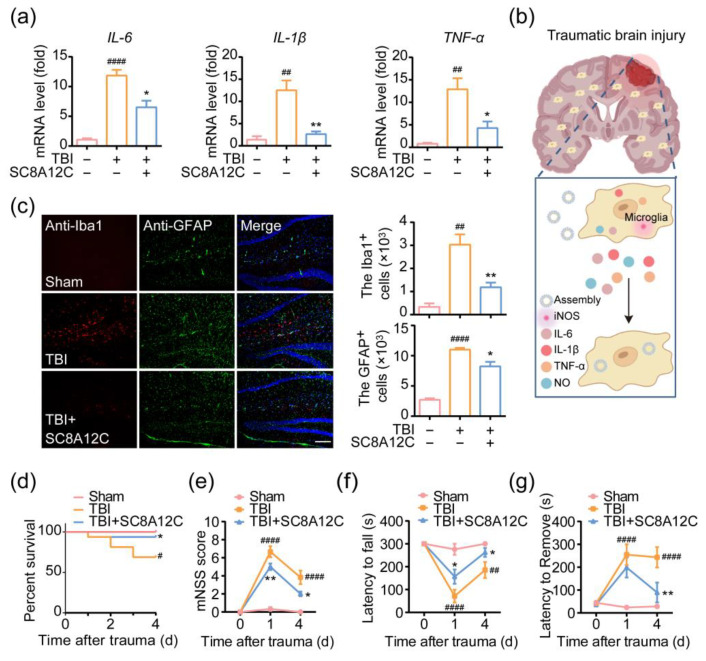
(**a**) RT-qPCR quantitative analysis of *IL-6*, *IL-1β*, *TNF-α* levels in the injured cortex at 1-day post-injury of sham mice, TBI mice with or without SC8A12C treatment (n = 3); (**b**) schematic diagram of SC8A12C treating TBI by inhibiting microglia activation; (**c**) fluorescence images and counting of Iba^+^ and GFAP^+^ cells in the penumbral regions of brain slices from sham, TBI, and TBI + SC8A12C groups (n = 3). Scale bar, 50 μm; (**d**) survival rates of sham mice, and TBI mice with or without SC8A12C administration (n = 6) during 4 days; (**e**) the mNSS test at 1 and 4 days post-injury in sham mice, TBI mice with or without SC8A12C administration (n = 6); (**f**) rotarod analysis of motor function at 1, 4 days post-injury in sham mice, TBI mice with or without SC8A12C administration (n = 6); (**g**) the adhesive tape removal test, the latency to remove for ipsilateral forepaw was evaluated at 1 and 4 days post-injury in sham mice, TBI mice with or without SC8A12C administration (n = 6). ^#^ *p* < 0.05, ^##^ *p* < 0.01, ^####^ *p* < 0.0001 vs. sham group; * *p* < 0.05, ** *p* < 0.01 vs. TBI group.

We also observed the attenuation of inflammatory mediators in the brain tissue of SC8A12C-treated TBI mice (Figure 6a). We analyzed the expression of inflammation-associated genes and found a significant increase in TBI mice. However, after SC8A12C treatment, the transcriptions of inflammation-associated genes were dramatically downregulated. As microglia and astrocytes are the main nerve cells that mediate the inflammatory response in the brain (Figure 6b), we then investigated the expression of microglia and astrocyte by labeling with the specific antibody of Iba1 (ionized calcium-binding adaptor molecule 1) for microglia and GFAP (glial fibrillary acidic protein) for astrocytes, respectively. Figure 6c showed higher Iba1 and GFAP immunoreactivity in TBI mice than that in the sham group, suggesting that strong neuroinflammation occurs after TBI. In contrast, the SC8A12C evidently decreased the Iba1 and GFAP immunoreactivity, revealing that SC8A12C effectively blocked TBI-mediated over-activation of microglia and astrocytes.

Finally, we recorded the survival status and behavioral abilities of the mice. We recorded the survival rate of mice in each group and observed the continuous occurrence of mouse death in the TBI group up to 4-d post-injury (Figure 6d). We observed the opposite trend in the presence of SC8A12C, which had a survival rate of over 90% after SC8A12C administration, suggesting that SC8A12C is a valuable agent for TBI therapy. Neurological function was estimated by the neurological severity score as described previously [30], presenting a great improvement of the neurological function in the SC8A12C-treated group as observed on day 1 and day 4 post-TBI (Figure 6e). The rotarod test is usually used to assess the motor coordination ability after TBI. Compared with TBI mice, the time to remain on the drum was significantly prolonged on day 1 and day 4 after SC8A12C administration in the TBI mice, proving the recovery of motor function (Figure 6f). The adhesion removal test is a sensitive method to evaluate sensory-motor defects in mice. Figure 6g indicates that mice in the SC8A12C-treated group removed the tape from their claws faster than that in the TBI group. Collectively, these findings suggest that single local administration of SC8A12C could effectively mitigate the sensory and motor deficiencies induced by TBI, enhancing sensory and motor function in the initial stage of TBI.

## 3. Discussion

### 3.1. Selection of Calixarenes

During the past few decades, calixarenes [31] have attracted increasing attention for their biomedical applications [32,33]. Calix[n]arenes are named according to the number of phenol residues in calixarenes, among which calixarenes, calix[4]arenes, calix[5]arenes, calix[6]arenes, and calix[8]arenes are the most widely studied. We employed four types of calixarenes to systematically explore the effect of calixarenes on neuroinflammatory inhibition. Finally, SC8A was selected as the preferred candidate due to its superior activity and biocompatibility. As the skeleton increases, the anti-inflammatory effect will become stronger. Calixarene with a flexible skeleton has a good anti-inflammatory effect, which is the basis of designing compounds with an anti-inflammatory effect.

Considering that the assemblies formed by calixarene amphiphilic molecules promote cell endocytosis, we introduced alkyl chains into SC8A to obtain SC8A12C. The critical micelle concentration of amphiphilic calixarene modified with a short alkyl chain is relatively large, however, the water-solubility of amphiphilic calixarene modified with a long alkyl chain is relatively poor. Therefore, we synthesized SC8A12C with an alkyl chain of appropriate length. In our study, we observed that SC8A12C presented micellar particles under TEM. Moreover, the anti-inflammatory effect of SC8A12C assemblies was better than that of SC8A. In summation, this study provides meaningful insight into the design of new supramolecular assemblies for exploiting their intrinsic activation and ability to serve as a drug carrier.

### 3.2. Endocytosis Mechanism of SC8A12C Assemblies

In previous research, we investigated and found that the internalization of the assemblies (GC5A-12C) was mediated by caveolin, which is a way of energy consumption [34]. In addition, Lalor and coworkers explored the endocytic pathway of a water-soluble calix[4]arene and demonstrated that clathrin or caveolin mediated endocytosis was not the main internalization mechanism of this calixarene [35]. To observe the accumulation of SC8A12C in cells, we tracked SC8A12C-PFBT (PFBT and SC8A12C were assembled into fluorescent probes to track nano-assemblies) using fluorescence imaging and studied the internalization pathway. We found that macropinocytosis played a crucial role in the uptake of SC8A12C assemblies in microglia cell line. The endocytosis pathway of calixarene may be related to cell type. Immune cells, such as macrophages and neutrophils, exhibit high levels of macropinocytosis when sampling substances from the extracellular environment [36,37,38]. We speculate that macropinocytosis is also an important way of phagocytosis of microglia, the resident immune cells in the brain. The cell uptake of SC8A12C loaded with PFBT also showed that the SC8A12C assemblies were capable of drug loading and drug delivery, which may add to their therapeutic possibility.

### 3.3. SC8A12C Penetrated into Brain Parenchyma through Local Administration in a Mouse Model of TBI

It is quite difficult for substances in the blood to enter the brain, due to the existence of the BBB, which is a dense barrier composed of endothelial cells and astrocytes [39,40]. However, intense mechanical injury leads to exposure of brain tissue [41]. TBI triggers a profound neuroinflammatory response, leading to the breakdown of the BBB [42]. Only local administration can be used for first aid, and this mode of administration does not require drugs to enter brain tissue through blood flow, which will avoid the difficulty of BBB penetration. In addition, local administration can also avoid the side effects caused by systemic administration. Thus, we administered SC8A12C with an eyedropper to the exposed brain tissue. We found that the assemblies penetrated into the brain parenchyma. We believed that this was also the prerequisite for calixarene to exert its anti-inflammatory effect in the TBI model.

### 3.4. In Vivo Therapeutic Effect of SC8A12C Assemblies on Traumatic Brain Injury

Traumatic brain injury leads to neuroinflammation mediated by microglia, which aggravates a series of injuries, such as BBB destruction and neurological dysfunction [43,44,45,46]. Surprisingly, common anti-inflammatory drugs, steroids, have failed results in clinical trials, which may be due to the lack of targeting of systemic administration. Therefore, it is necessary to develop new treatments targeting neuroinflammation mediated by microglia. Our result showed that SC8A12C exhibited an excellent ability to eliminate inflammatory factor storms in a mouse model of TBI, thereby restoring neurobiological functions and alleviating TBI-induced sensory and motor deficiencies. Our work provides a new perspective for the design of molecules with anti-neuroinflammatory activities. This research has led to some important research directions, such as the mechanism of calixarene or calixarene assemblies inhibiting neuroinflammation. Perhaps the structure of calixarene (including the skeleton and conformation) affects ion channels or epigenetic factors. The study of underlying mechanisms is important but involves a complex, strict, and time-consuming process. We have explored whether SC8A12C affects the TLR4-related neuroinflammatory signaling pathway but did not observe any effect of SC8A12C on TLR4 protein expression (Figure S9). In the future, we will thoroughly study the anti-neuroinflammatory mechanism of calixarenes.

## 4. Materials and Methods

### 4.1. Materials

SC4A, SC5A, SC6A, SC8A, and SC8A12C were synthesized according to previous literature [47,48]. Synthesis of SC8A12C: Briefly, 3.14 g of NaOH and 19.66 g of 1-dodecyl bromide were dissolved in 15 mL of water and 60 mL of dimethyl sulfoxide, respectively. At this time, SC8A (3.20 g, 1.92 mmol) was mixed with NaOH and 1-dodecylbromide solutions, and the resulting mixture was heated at 50 °C for 24 h. After the reaction, the product was precipitated from the solution with methanol and the precipitate was dissolved in 10 mL of water. The resulting solution was precipitated again with ethanol. The above steps were repeated three times to remove NaBr. The yield of SC8A12C was 83%. The ^1^H NMR (400 MHz, dimethyl sulfoxide [DMSO]-d6, δ) results were as follows: 7.26 (s, 16H; ArH), 4.29 (d, J = 15.8 Hz, 8H; Ar-*CH*_2_-Ar), 3.61 (m, 24H; -*CH*_2_-O-Ar and Ar-*CH*_2_-Ar), 1.64 (s, 16H; -*CH*_2_-CH_2_-O-Ar), 1.53–1.02 (m, 144H; alkyl CH_2_), 0.84 (s, 24H; *CH*_3_-CH_2_-). Preparation of SC8A12C solution: 2.5 mg of SC8A12C was dissolved in 1 mL of water, and the resulting solution was ultrasonicated at 80 °C for 1 h.

SC8A12C-PFBT solution was synthesized according to previous literature [49]. Furthermore, 100 μL of SC8A12C solution (0.5 mg/mL) and 2.5 μL of DSPE-PEG2000 solution (1 mg/mL) were diluted in 2 mL of water, the resulting mixture 1 was ultrasonicated at 80 °C for 9 min. Furthermore, 25 μL of PFBT solution (1 mg/mL) was diluted in 250 μL of tetrahydrofuran, and 200 μL of the resulting solution was added under ultrasonication at 80 °C. The final reaction solution was cooled to room temperature, filtered through a filter (220 nm), and the tetrahydrofuran was removed in vacuo.

3-(4,5-Dimethyl-2-thiazolyl)-2,5-diphenyl-2-*H*-tetrazolium bromide (MTT, M8180), 0.01 M phosphate-buffered saline (PBS, powder, pH7.2-7.4), 4′,6-diamidino-2-phenylindole (DAPI), and 2′-(4-hydroxyphenyl)-5-(4-methyl-1-piperazinyl)-2,5′-bi-1H-benzimidazole trihydrochloride hydrate (Hoechst, 33258) were purchased from Beijing Solarbio Science & Technology Co., Ltd. (Beijing, China). Dulbecco’s modified Eagle medium (DMEM), and penicillin streptomycin were purchased from Thermo Fisher Scientific (Waltham, MA, USA). Amiloride and nystatin were purchased from Sigma (St Louis, MO, USA). Chlorpromazine was purchased from Toronto Research Chemicals (Toronto, ONT, Canada).

### 4.2. Characterization of SC8A12C Assemblies

The dynamic light scattering (DLS) technique (Zetasizer Nano ZS, Malvern, UK) was used to detect the average particle size and potential of SC8A12C assemblies. The sample concentration was kept at 25 μg/mL. The data were collected and analyzed by software (v7.12). The dispersant was deionized water. The refractive index and viscosity of the dispersant were 1.330 and 0.8872, respectively. The dispersant dielectric constant was 78.5. The refractive index of samples was 1.59. The instrument is equipped with a 633 nm red laser. The incident light source adopts a He-Ne laser light source, the scattering angle is 173°, and the temperature is 25 °C.

The morphology of the SC8A12C assemblies was examined under a transmission electron microscope (Talos F200C, Thermo, Waltham, MA, USA) and an atomic force microscope (Dimension Icon, Bruker, Bilerica, MA, USA). Samples were placed on carbon-coated copper grids, stained with phosphotungstic acid and dried for transmission electron microscope (TEM) observation. The sample for AFM measurement was prepared by dropping the sample solution onto a silicon pellet and then dried.

### 4.3. Stability of SC8A12C Assemblies

The stabilities of SC8A12C assemblies in buffers with different pH values and temperatures were studied by monitoring the change in particle size. SC8A12C assemblies were suspended in pH 5, pH 6, pH 7, pH 8, and pH 9 buffers at room temperature with gentle agitation for 2 h, and the particle sizes were measured by DLS. To identify the effect of temperature on the assemblies, SC8A12C assemblies were suspended in ddH_2_O. The suspensions were incubated at 25 °C, 50 °C, and 75 °C for 30 min and determined.

### 4.4. In Vitro Cytotoxicity Assay

One hundred microliters of a uniform BV2 cell suspension were added to a 96-well plate. After the cell adhered to the plate wall, the control group was treated with DMEM (Gibco, Waltham, MA, USA) only, and the administration groups were treated with SC4A, SC5A, SC6A, SC8A, SC8A12C, and SC8A12C-PFBT for 24 h. After 24 h, the medium was discarded, 1 mg/mL MTT was added, and the cells were incubated at 37 °C for 4 h. The medium was then discarded, 150 μL of DMSO was added, and the cells were shaken for 10 min. The microplate reader was used to detect the absorbance of 570 nm.

### 4.5. Measurement of NO Production

One hundred microliters of a uniform BV2 cell suspension were added to a 96-well plate. After the cell adhered to the plate wall, they were treated for 24 h with either calixarene plus LPS (0.1 μg/mL) or LPS (L4516, Sigma) alone. Griess reagent was used to detect NO in the cell culture medium. In brief, at the treatment endpoint, 50 μL of the medium was collected, to which 50 μL of Griess reagent was added. After mixing, the microplate reader was used to detect the absorbance of 540 nm.

### 4.6. Western Blot

Two milliliters of a uniform BV2 cell suspension were added to a 6-well plate. After the cell adhered to the plate wall, they were treated for 24 h with either calixarene plus LPS (0.1 μg/mL) or LPS (L4516, Sigma) alone. The harvested cell samples were lysed in lysis buffer and the protein concentration was determined in each sample using a Bradford assay kit (Beyotime, p0006). The samples were separated by sodium dodecyl sulfate–polyacrylamide gel electrophoresis, transferred to 0.45 μm polyvinylidene difluoride membranes (Merck, Kenilworth, NJ, USA; IPVH15150), and incubated with primary antibodies against iNOS (Cell Signaling Technology, Danvers, MA, USA; 13120) or β-tubulin (Easy Bio, Seoul, Korea; BE0025).

### 4.7. Animal

Adult male C57BL/6 mice were purchased from Beijing Vital River Laboratory Animal Technology Co., Ltd. (Beijing, China) and housed in the Experimental Animal Center of Nankai University. At 6–8 weeks, the mice were divided into sham, TBI, and TBI + SC8A12C groups. The mice were anesthetized and placed on a stereotactic frame. Their heads were shaved and disinfected and a 2 cm incision was made in the middle of the scalp. The left parietal periosteum was removed and fixed on the mouse brain stereotactic instrument at coordinates of AP 1.0 mm, lat 1.0 mm, and DEP 2.5 mm. A 4 mm diameter hole was drilled in the left parietal bone. Using the free-fall method, a 40 g counterpoise was dropped from a height of 3 cm and hit a cylinder on the surface of the endocranium. The diameter of the cylinder was 4 mm. When the cylinder was impacted, it dropped 1 mm. Thus, the TBI model was established. The wound was disinfected layer by layer to prevent infection and the scalp was sutured. Mice in the TBI + SC8A12C group received a SC8A12C solution (10 μL, 2.5 mg/mL) and mice in the sham group received double-distilled (dd) H_2_O (10 μL), which were slowly added to the brain wound before the scalp was sutured.

### 4.8. Brain Imaging

Ten microliters of ddH_2_O (sham and TBI groups) or ten microliters of SC8A12C-PFBT (TBI group) were administered locally via an eyedropper. Each mouse was euthanized and their brains were dissected. The IVIS in vivo imaging system (PerkinElmer, Waltham, MA, USA) was used for brain imaging. SC8A12C-PFBT was detected using an excitation wavelength of 460 nm and an emission wavelength of 520 nm. IVIS data were processed using the Living Image software version 4.5.5. (PerkinElmer). The radiant efficiency ((p/s)/(μW/cm^2^)) was determined in a circular region of interest (ROI). A 1.5-cm diameter ROI was used for brain measurement.

### 4.9. Rotarod Test

In brief, the mouse was placed on a rotating cylinder and accelerated to 25 rpm over a period of 5 min. The time taken for the mouse to fall off the drum was recorded. Five days before the experiment, the mice were trained and those that failed to meet the training standard were excluded. Then, the rotarod test was officially implemented at 1-day post-TBI.

### 4.10. Neurological Severity Score (NSS) for Mice

Mice were subjected to a neurological assessment using a 10-point scale according to the Garcia neurological grading criteria [50].

### 4.11. Adhesive Removal Test

Sensory-motor deficits were assessed using an adhesion removal test. The mice were kept in a transparent beaker and allowed to be adapted for 30 min. An adhesive tape was applied to the hairless part of the front paw and the mice were placed back into the beaker. The time taken to remove the adhesive tape was recorded. The maximum time allowed for this experiment was 300 s.

### 4.12. Identification of Proinflammatory Factor Gene Transcription Level

The total RNA was extracted from BV2 cells or brain tissue with Trizol (Invitrogen, Carlsbad, CA, USA; 15596026). cDNA was then reverse transcribed from total RNA using a TransScript First-Strand cDNA Synthesis kit (Transgen, Beijing, China; AT301-02). Realtime polymerase chain reaction (qPCR) was performed using the QuantStudio 5 Real-Time PCR System (Life Technologies, Carlsbad, CA, USA) with SYBR Green reagent (TIANGEN, Beijing, China; FP205-02) to quantify the expression levels of inflammation-related genes. The PCR primer sequences are listed in Table 1.

### 4.13. Fluorescence Imaging at the Cellular Level

To assess cell internalization, BV2 cells were seeded on confocal dishes and incubated with SC8A12C-PFBT (25 μg/mL) at 37 °C for 4 h, 12 h, and 24 h, respectively. The medium was discarded and the cells were washed with PBS, fixed in 4% paraformaldehyde, and permeabilized with 0.1% Triton X-100. Finally, the nuclei were stained with DAPI for 10 min. A confocal imaging system (Leica Wetzlar, Germany; SP8) was used to capture fluorescence images.

To investigate the cellular uptake mechanism of the SC8A12C assemblies, BV2 cells were seeded in confocal dishes. After the cell adhered to the dish walls, three inhibitors: amiloride (0.01 μM), chlorpromazine (5 μM), and nystatin (10 μM) were added separately. After 60 min of incubation, SC8A12C-PFBT (25 μg/mL) was added to the cell culture medium and the cells were cultured at 37 °C for 12 h. The medium was discarded and the cells were washed with PBS and then fixed in 4% paraformaldehyde. Finally, the cell membrane was stained with WGA Texas Red (2086755, Invitrogen, Carlsbad, CA, USA) and the nucleus was stained with Hoechst 33342. Cellular uptake of SC8A12C-PFBT in BV2 was observed in vitro by a confocal microscope (Leica Wetzlar, Germany, SP8).

For immunofluorescence, BV2 cells were cultured on confocal dishes and incubated with SC8A12C-PFBT plus LPS (0.1 μg/mL) or LPS alone at 37 °C for 24 h. The medium was discarded and the cells were washed with PBS and then fixed in 4% paraformaldehyde, and permeabilized with 0.1% Triton X-100. The cells were then incubated with anti–iNOS primary antibody (1:200, Cell Signaling Technology, 13120) overnight at 4 °C. The primary antibody was discarded, and the cells were washed with PBS and then incubated with the secondary antibody (tetramethylrhodamine-conjugated goat anti-rabbit IgG; 1:200; Jackson ImmunoResearch, West Grove, PA, USA) for 1 h at room temperature. The nuclei were stained with DAPI for 10 min. Images of the co-localization of SC8A12C-PFBT and iNOS were captured using a confocal imaging system (Leica SP8).

### 4.14. Evans Blue Staining

Evans blue staining was used to assess the integrity of the BBB. Briefly, 24 h after TBI, the mice were intravenously injected with 2% Evans blue dye (Sigma-Aldrich, St. Louis, MI, USA; 6 mL/kg in saline) and the dye was allowed to circulate in the body for 2 h. The leakage of Evans blue into the injured brain tissue was analyzed. The whole brain was rapidly harvested and weighed. The dye was extracted from brain tissue and the absorbance was measured at 620 nm using a spectrophotometer.

### 4.15. Statistical Analysis

Data were expressed as mean ± standard error of the mean (SEM). Statistical analysis was performed with GraphPad Prism software (version 6.0; GraphPad, San Diego, CA, USA). A one-way analysis of variance (ANOVA) was used to assess significant differences between multiple groups, and a post hoc comparison was performed using Sidak’s multiple comparisons test. Survival rates were compared between groups using a log-rank (Mantel–Cox) test. For the NSS, rotarod test, and adhesion removal test, a two-way ANOVA was used to assess significant differences between multiple groups, with post hoc comparison performed using Tukey’s multiple comparisons test. Significant differences were indicated when *p* < 0.05.

## Figures and Tables

**Table 1:** Primer sequences.

ID	Primer Sequence
*β-Actin*	Forward:5′-GGCTGTATTCCCCTCCATCG-3′
	Reverse:5′-CCAGTTGGTAACAATGCCATGT-3′
*m-IL-6*	Forward:5′-AGCCAGAGTCCTTCAGAGAG-3′
	Reverse:5′-CTTAGCCACTCCTTCTGTGAC-3′
*m-IL-1β*	Forward:5′-TGTGTAATGAAAGACGGCAC-3′
	Reverse:5′-TCCACTTTGCTCTTGACTTC-3′
*m-TNF-α*	Forward:5′-CAAAATTCGAGTGACAAGCCT-3′
	Reverse:5′-CTGGGAGTAGACAAGGTACAAC-3

## Data Availability

The data presented in this study are available in this article.

## Supplementary Materials

The following supporting information can be downloaded at: https://www.mdpi.com/article/10.3390/molecules27092967/s1. Figure S1 Structure of SC8A12C; Figure S2 ^1^H NMR spectrum of SC8A12C in DMSO d6, 400 MHz, 25 °C; Figure S3 Viabilities of BV2 cells treated with SC8A12C-PFBT for 24 h. Cells cultured without SC8A12C-PFBT served as the control group. Cell viabilities were measured by MTT assay and are shown as the percentage of untreated cells. Data presented as mean ± SEM, *n* = 7. p-values are calculated by using one-way analysis of variance (ANOVA) with Sidak’s multiple comparisons test, **** *p* < 0.0001 vs. the control group; Figure S4 Measurement of NO generation by Griess assay. BV2 cells were treated with LPS (0.1 μg/mL) alone, LPS (0.1 μg/mL) with SC8A12C-PFBT for 24 h. Cells cultured without LPS (0.1 μg/mL) and SC8A12C-PFBT served as the control group. Data presented as mean ± SEM, *n* = 3. *P*-values are calculated by using one-way analysis of variance (ANOVA) with Sidak’s multiple comparisons test, ^####^ *p* < 0.0001 vs. the control group; *** *p* < 0.001 vs. the LPS group; Figure S5 Representative (a) and quantitative (b) Western blot assay results, in which the lanes represent the iNOS or β-Tubulin level in cell sample as shown above. Data presented as mean ± SEM, *n* = 2. p-values are calculated by using one-way analysis of variance (ANOVA) with Sidak’s multiple comparisons test, ^#^ *p* < 0.05 vs. the control group; ** *p* < 0.01 vs. the LPS group; Figure S6 RT-qPCR quantitative analysis of *IL**-6* level in BV2 cells treated with LPS (0.1 μg/mL) alone, LPS (0.1 μg/mL) with SC8A12C-PFBT for 24 h. Data presented as mean ± SEM, *n* = 3. p-values are calculated by using one-way analysis of variance (ANOVA) with Sidak’s multiple comparisons test, ^####^ *p* < 0.0001 vs. the control group; **** *p* < 0.0001 vs. the LPS group; Figure S7 RT-qPCR quantitative analysis of *IL**-1β* level in BV2 cells treated with LPS (0.1 μg/mL) alone, LPS (0.1 μg/mL) with SC8A12C-PFBT for 24 h. Data presented as mean ± SEM, *n* = 3. p-values are calculated by using one-way analysis of variance (ANOVA) with Sidak’s multiple comparisons test, ^####^ *p* < 0.0001 vs. the control group; **** *p* < 0.0001 vs. the LPS group; Figure S8 RT-qPCR quantitative analysis of *TNF**-α* level in BV2 cells treated with LPS (0.1 μg/mL) alone, LPS (0.1 μg/mL) with SC8A12C-PFBT for 24 h. Data presented as mean ± SEM, *n* = 3. p-values are calculated by using one-way analysis of variance (ANOVA) with Sidak’s multiple comparisons test, ^####^ *p* < 0.0001 vs. the control group; * *p* < 0.05 vs. the LPS group; Figure S9 Representative (a) and quantitative (b) Western blot assay results, in which the lanes represent the TLR4 or GAPDH level in cell sample as shown above. Data presented as mean ± SEM, *n* = 3. No effect of SC8A12C on TLR4 protein expression was observed.

## Sample Availability

Samples of the compounds are available from the authors.
