# Supplementary material for: A Calixarene Assembly Strategy of Combined Anti-Neuroinflammation and Drug Delivery Functions for Traumatic Brain Injury Therapy
Source: Molecules. 2022 May 6;27(9):2967. doi: 10.3390/molecules27092967 (PMC9101726; doi:10.3390/molecules27092967)
Supplement: Supplementary file 1 [file molecules-27-02967-s001.zip › molecules-1654368-supplementary.pdf]

Supporting Information

A calixarene assembly strategy of combined the anti-neuroinflammation and drug delivery functions for traumatic brain injury therapy

Chunxiao Wang[1], Yu-Xuan Chang[2], Xi Chen[1], Lihuan Bai[1], Heping Wang[1], Yu-Chen Pan[2], Chunqiu Zhang[1], Dong-Sheng Guo[2]*, Xue Xue[1]*

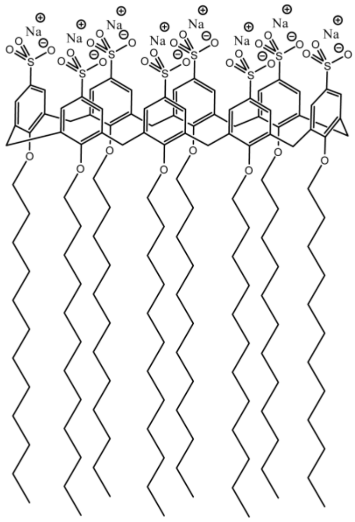

Figure S1 Structure of SC8A12C.

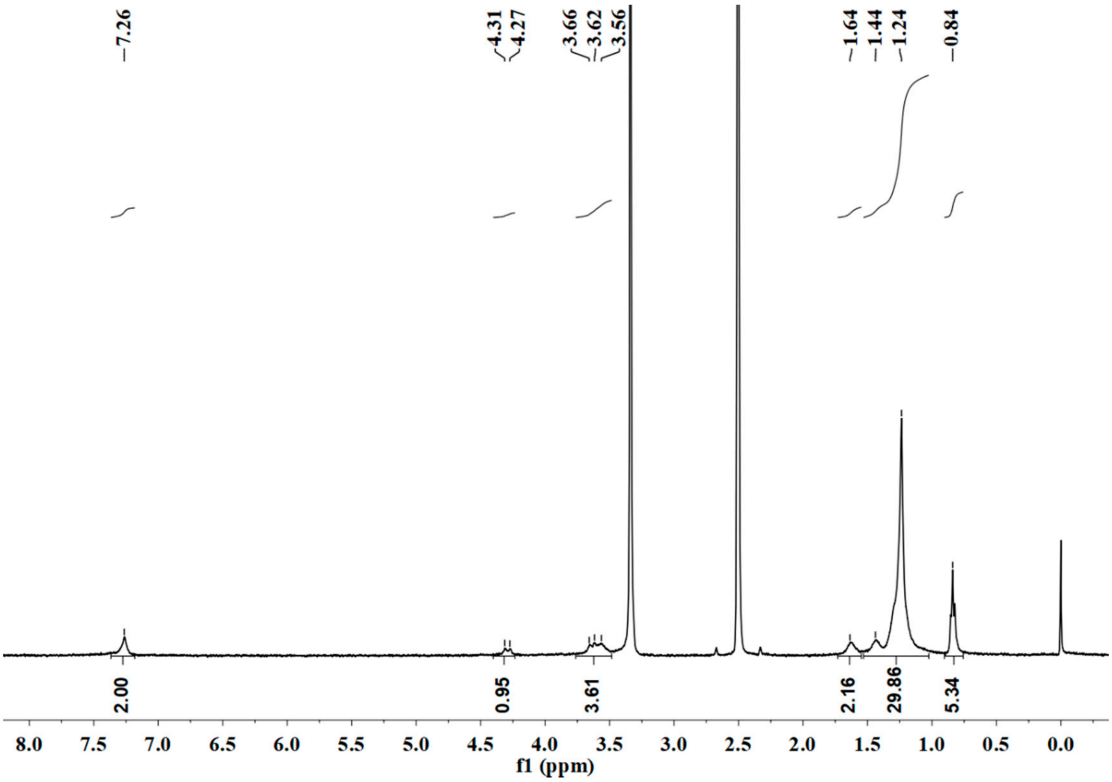

Figure S2. ^1H NMR spectrum of SC8A12C in DMSO-d_6, 400 MHz, 25 °C.

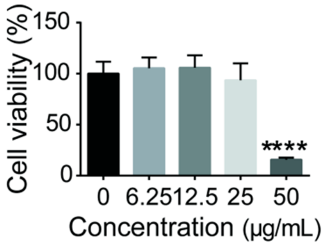

Figure S3. Viabilities of BV2 cells treated with SC8A12C-PFBT for 24 h. Cells cultured without SC8A12C-PFBT served as the control group. Cell viabilities were measured by MTT assay and are shown as the percentage of untreated cells. Data presented as mean ± SEM, n = 7. p-values are calculated by using one-way analysis of variance (ANOVA) with Sidak's multiple comparisons test, ****p < 0.0001 vs the control group.

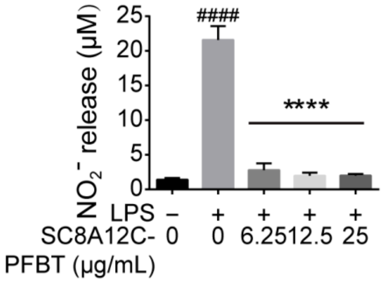

Figure S4. Measurement of NO generation by Griess assay. BV2 cells were treated with LPS (0.1 μg/mL) alone, LPS (0.1 μg/mL) with SC8A12C-PFBT for 24 h. Cells cultured without LPS (0.1 μg/mL) and SC8A12C-PFBT served as the control group. Data presented as mean ± SEM, n = 3. p-values are calculated by using one-way analysis of variance (ANOVA) with Sidak's multiple comparisons test, ####p < 0.0001 vs the control group; ***p < 0.001 vs the LPS group.

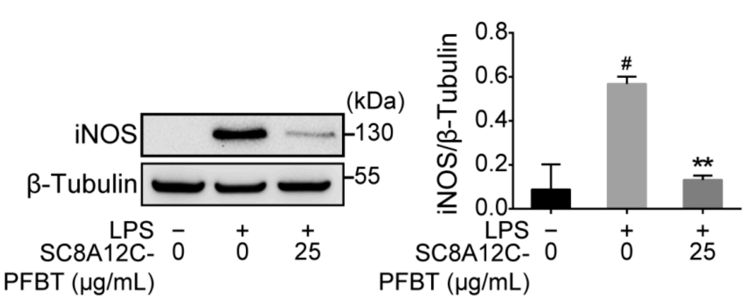

Figure S5. Representative (a) and quantitative (b) Western blot assay results, in which the lanes represent the iNOS or β-Tubulin level in cell sample as shown above. Data presented as mean ± SEM, n = 2. p-values are calculated by using one-way analysis of variance (ANOVA) with Sidak's multiple comparisons test, #p < 0.05 vs the control group; **p < 0.01 vs the LPS group.

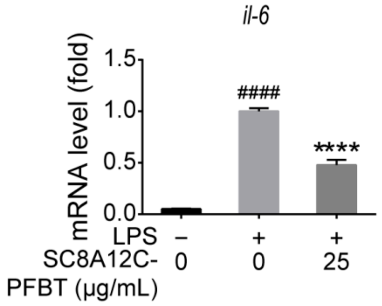

Figure S6. RT-qPCR quantitative analysis of *il-6* level in BV2 cells treated with LPS (0.1 μg/mL) alone, LPS (0.1 μg/mL) with SC8A12C-PFBT for 24 h. Data presented as mean ± SEM, n = 3. p-values are calculated by using one-way analysis of variance (ANOVA) with Sidak's multiple comparisons test, ####p < 0.0001 vs the control group; ****p < 0.0001 vs the LPS group.

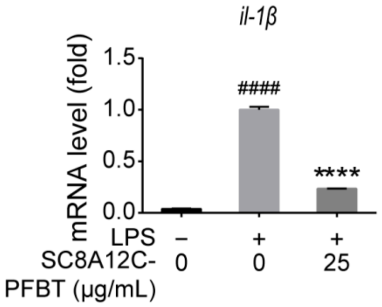

Figure S7. RT-qPCR quantitative analysis of *il-1β* level in BV2 cells treated with LPS (0.1 μg/mL) alone, LPS (0.1 μg/mL) with SC8A12C-PFBT for 24 h. Data presented as mean ± SEM, n = 3. p-values are calculated by using one-way analysis of variance (ANOVA) with Sidak's multiple comparisons test, ####p < 0.0001 vs the control group; ****p < 0.0001 vs the LPS group.

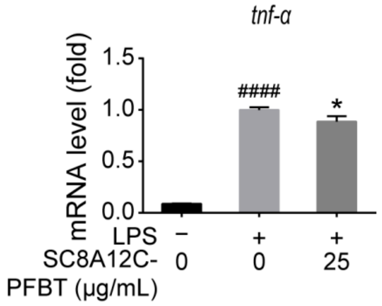

Figure S8. RT-qPCR quantitative analysis of *tnf-α* levels in BV2 cells treated with LPS (0.1 μg/mL) alone, LPS (0.1 μg/mL) with SC8A12C-PFBT for 24 h. Data presented as mean ± SEM, n = 3. p-values are calculated by using one-way analysis of variance (ANOVA) with Sidak's multiple comparisons test, ####p < 0.0001 vs the control group; *p < 0.05 vs the

LPS group.

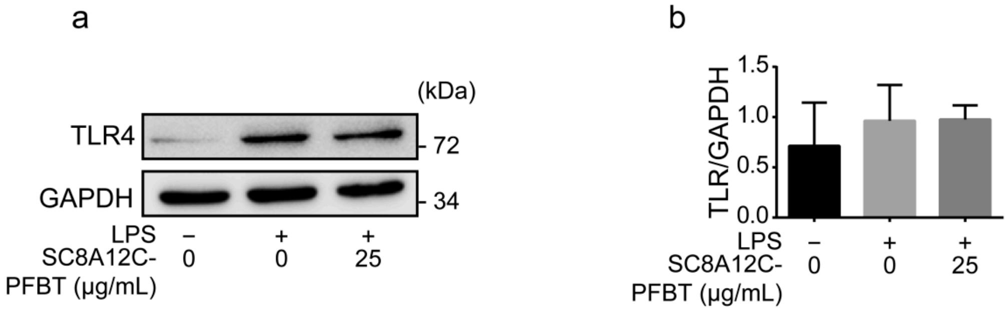

Figure S9. Representative (a) and quantitative (b) Western blot assay results, in which the lanes represent the TLR4 or GAPDH level in cell sample as shown above. Data presented as mean ± SEM, n = 3. No effect of SC8A12C on TLR4 protein expression was observed.
